# Continuously sustained elimination of iodine deficiency: a quarter of a century success in the Islamic Republic of Iran

**DOI:** 10.1007/s40618-018-0838-8

**Published:** 2018-02-14

**Authors:** H. Delshad, P. Mirmiran, Z. Abdollahi, F. Salehi, F. Azizi

**Affiliations:** 1 Micronutrient Research Office, Research Institute for Endocrine Sciences, Shahid Beheshti University of Medical Sciences, 19395-4763 Tehran, Islamic Republic of Iran; 2 Nutrition Research Center, Research Institute for Endocrine Sciences, Shahid Beheshti University of Medical Sciences, Tehran, Islamic Republic of Iran; 3 Ministry of Health and Medical Education, Tehran, Islamic Republic of Iran; 4 Research Institute for Endocrine Sciences, Shahid Beheshti University of Medical Sciences, Tehran, Islamic Republic of Iran

**Keywords:** Iodine, Iodine deficiency, UIC, Iodized salt, Iran

## Abstract

**Background:**

Iodine deficiency and related disorders were very common in Iran prior to 1996, when universal salt iodization (USI) was implemented and in 2000 Iran was declared iodine deficiency disorders (IDD) free. The aim of this study was to evaluate the adequacy of iodine intake by Iranian households in all 30 provinces of Iran, a quarter of a century after the intervention.

**Methods:**

A total of 18,000 school-aged children (8–10 years with mean 8.7 ± 1 year) were included in this study. Urine samples were collected from all children for measurement of urinary iodine excretion and 1800, 210 and 3000 salt samples were randomly collected from the family kitchen, production site of 73 salt factories and distribution circles of 30 provinces, respectively.

**Results:**

The median urinary iodine concentration (UIC) of participants was 161 μg/L. The proportion of children with UIC of, 20–49, 50–99 and ≥ 100 μg/L were 10.3, 15.9 and 73.7%, respectively. The mean (± SD) and median salt iodine values were 28.2 (± 12.6) and 31.7 ppm, at the production site, and 31.5 (± 13.6) and 29.6 ppm at the distribution circles, respectively. About 80% of factory salts had more than 20 ppm iodine. 98% of households consumed iodized salt, 80% had appropriate salt storage, and 83% of the household salts contained ≥ 20 ppm.

**Conclusions:**

Based on the results of this study, Iranian populations are consuming adequate iodine. The well-maintained and monitored USI program has improved the dietary iodine intakes of the population, and the country has achieved all criteria of a well-controlled IDD program.

## Introduction

As an essential nutrient, iodine is required for the production of thyroid hormones [1]. Since the body does not make iodine, it relies on the diet for sufficient iodine intake. The main cause of iodine deficiency is low iodine content in the diet. Severe iodine deficiency has adverse effects on the mean intelligence quotient of the population [2], and iodine supplementation could increase the intelligent quotient in children affected by iodine deficiency [3]. Low iodine intake is the main cause of preventable mental retardation. Worldwide efforts by international agencies have resulted in achieving iodine sufficiency by the year 2005 [4–7], although many countries, even in industrialized ones, are still iodine deficient [8–12]. Universal salt iodization as a safe, cost-effective and sustainable strategy was recommended by the World Health Organization (WHO) and the United Nations Children^’^s Fund (UNICEF) in 1994 to ensure sufficient intake of iodine by all individuals. Over the last century, considerable efforts worldwide have been paid to control this nutritional problem, but still many countries in the world are iodine deficient. Globally, 29.8% of school-age children (246 million) are estimated to have insufficient iodine intake. During the past decade, the number of iodine-deficient countries has decreased from 54 to 30 while iodine-sufficient ones have increased from 67 to 112; the number of countries with excessive iodine intake has increased from 5 to 10. Although worldwide 90% of households consume adequately iodized salt, consumption is still below 50% in 39 countries [13–15].

In Iran, iodine deficiency was common in most of its regions, with moderate to severe endemic goiter, cretinism, retarded brain and mental development being common in many parts of the country [16, 17]. Deficiency in iodine nutrition, despite being recognized in Iran since 1968 [18], was not categorized as a public health problem until 1980. The National Iranian Committee for Control of IDD was formed in 1989 and since then USI has resulted in sustainable preventive programs of IDDs, leading to great success in IDD control and elimination. In 2000, IR Iran was recognized as an iodine-sufficient country by the WHO Eastern Mediterranean Regional Office [19].

The sustainability of iodine sufficiency is a major concern after achieving the criteria of iodine repletion [20, 21]. A false sense of iodine sufficiency of the population is the major cause of failure in iodine deficiency elimination programs [22]. The National Iranian Committee for Control of IDD has scheduled control programs every 5–6 years to evaluate the sustainability of the program. This study aimed at confirming the updated data on iodine nutrition among schoolchildren in the I.R. Iran.

## Materials and methods

A cross-sectional cluster survey among schoolchildren aged 8–10 years was conducted between October 2013 and February 2014, using recommended standard methods and approaches. Urinary iodine levels and the amount of iodine content of salt were measured among schoolchildren, at factories, distribution sites and households. The survey protocol was reviewed and approved by the ethics committee of the Research Institute of Endocrine Sciences (RIES) affiliated to Shahid Beheshti University of Medical Sciences.

## Subjects and sampling

### Urinary iodine concentration

For urinary iodine determination, 600 subjects, an equal number of girls and boys, i.e., 30 clusters of schoolchildren (*n* = 20each) aged 8–10 years were selected in each province. Written informed consent was received from the parents of participants before the study. Thirty primary schools were selected proportionate to population size [23] in each province from the national database maintained by the Ministry of Education; and 20 children, aged 8–10 years, were sampled at random in each school. From 30 provinces of the country, urine samples of overall 18,000 schoolchildren (equal numbers of rural and urban) were obtained. These samples were transferred in screw-top plastic bottles on ice to the RIES laboratory and kept frozen at – 20 °C until the time of iodine measurement at the end of the study. All participants were requested to bring a salt sample from their house, with the brand name written on it.

From salt factories, five samples from different parts of each of the 73 iodized salt producing factories and 100 samples from distribution sites in each province were collected and sent to the food and drug control laboratory of the health center in each province. At the measurement site, a small portion of the salt was tested with a rapid test kit (MBI Kits, India). Salt envelopes were marked with the subjects’ unique codes and samples were delivered to the laboratory of the Research Institute for Endocrine Sciences for measuring the iodine content by titration. Quantitative iodine measurement was performed at the center. Samples of iodized salt for household use (1800 samples from all provinces) were collected for quality and quantity control, and the content of household salt was measured in the field, using rapid testing kits [24]. Approximately, 10% of salt samples were randomly selected and transferred to the laboratory for food and drug control of the health center in each province for iodometric titration.

### Laboratory measurement

Three trained technicians measured the iodine concentration of all urine samples using the acid digestion method at the RIES laboratory, [25, 26]. The intra-assay coefficient of variation (CV) of the UIC measurement method for concentrations of 3.5, 15 and 38 µg/L was 11.2, 8.2 and 9.4%, respectively, and the inter-assay CV values for these concentrations were 12.5, 8.9 and 10.3%, respectively. Iodometric titration assay was used for quantitative salt iodine measurements [27] and values are shown in parts per million (ppm). The reaction mechanism for iodometric titration includes two steps. (1) Liberation of free iodine from salt by the addition of H_2_SO_4_ liberates free iodine from the iodate in the salt sample. Then, excess KI is added to help solubilize the free iodine, which is quite insoluble in pure water under normal conditions. (2) Titration of free iodine with thiosulfate. Free iodine is consumed by sodium thiosulfate in the titration step. The amount of thiosulfate used is proportional to the amount of free iodine liberated from the salt. Starch is added as an external (indirect) indicator of this reaction and reacts with free iodine to produce a blue color. When added toward the end of the titration, i.e., when only a trace amount of free iodine is left, the loss of blue color, or end point, which occurs with further filtration, indicates that all remaining free iodine has been consumed by thiosulfate. This titration method has been standardized by RIES and approved by the Ministry of Health and Medical Education (MHME) for uniform measurement of salt iodine in the laboratories for food and drug control of the health center of each province.

### Definitions

Iodine deficiency was considered as a median urinary iodine concentration (UIC) < 100 µg/L. Median UICs of 50–99, 20–49 and < 20 µg/L were considered mild, moderate and severe iodine deficiency, respectively, while median UICs of 100–199, 200–299 and ≥ 300 µg/L were considered adequate, more than adequate and excessive, respectively [28]. For salts, iodine levels of < 20, 20–40 and > 40 ppm were considered inadequate, adequate and excessive, respectively.

### Statistical analyses

Percentage, arithmetic mean, median and standard deviation were used to present the data. Appropriate tests of significance (*χ*^2^, Student’s test, analysis of variance (ANOVA) and Mann–Whitney *U* tests) were applied wherever necessary. Correlations between continuous numerical variables were assessed by Spearman’s rank and Pearson coefficients. SPSS 9.05 software package (SPSS, Inc., Chicago, IL) was used for the statistical analysis and *p* < 0.05 was considered to be significant.

## Results

A total of 18,000 school-aged children (8–10 years with mean 8.7 ± 1 year) were included in this study.

### Urinary iodine concentration

**Fig. 1:**
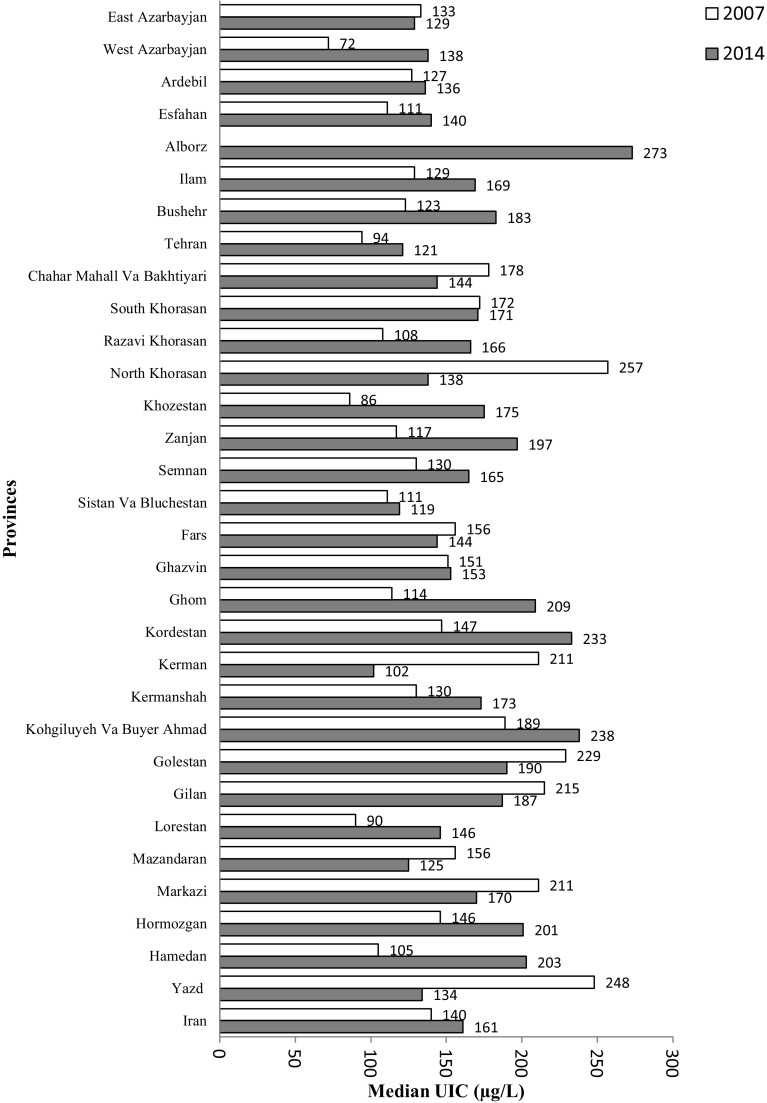
Comparison of median UICs in all provinces of Iran in 2007 and 2014

The median UIC of schoolchildren was 161 μg/L. Figure 1 shows the median UICs of all provinces in bar graphs compared to the previous study [29]; 10.3, 15.9 and 73.7% of children had urinary iodine excretion levels of 20–49, 50–99 and > 100 μg/L, respectively. There was no significant difference in UICs between boys and girls or between rural and urban areas (Table 1). All provinces had median UIC > 100 μg/L.

**Table 1:** Median urinary iodine content (UIC = µg/L) among schoolchildren aged 8–10 years of 30 provinces of Iran (*n* = 18,000)

	Girls	Boys	Total
Urban	163^a^	161	164
Rural	153	163	158
Total	158	162	161

^a^µg/L

### Salt iodine at the production level

The mean (± SD) and median salt iodine at the production level were 28.2 (± 12.6) and 31.7 ppm, respectively. Iodine levels of < 20, 20–40 and > 40 ppm were observed in 9, 71 and 20% of the samples, respectively. About 80% of factory salts had > 20 μg/L iodine.

### Salt iodine at the distribution level

The mean (± SD) and median of iodine level concentrations were 31.5 (± 13.6) and 29.6 ppm, respectively, at the distribution level. Iodine levels of < 20, 20–40 and > 40 ppm were observed in 11, 59 and 30% of the samples, respectively.

### Salt iodine at the household level

**Fig. 2:**
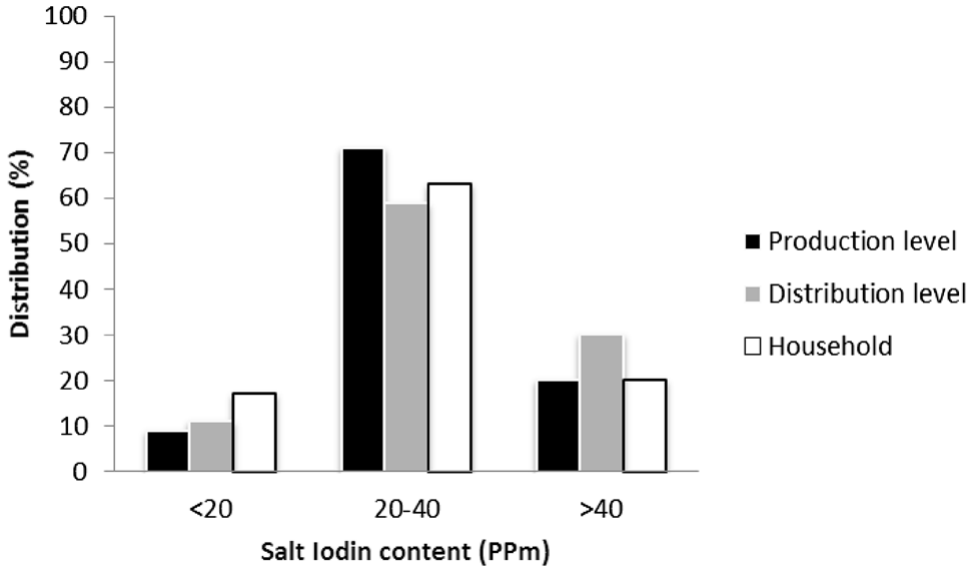
Distribution (%) of salt iodine content (ppm) at production, distribution and household levels (*ppm* parts per million)

Ninety-eight percent of households consumed iodinated salt, of which 82% was crystallized iodized salt. Fifty-eight percent of households had appropriate salt storage. Quantitative assays of household salt samples showed that the median iodine content was 30 ppm. Iodine levels < 20, 20–40 and > 40 ppm were observed in 17, 63 and 20% of household salts, respectively. An iodine content ≥ 40 ppm was more frequently found in the salt samples collected in provinces where the median UIC values in schoolchildren were more than 200 µg/L (76 VS 24%). Figure 2 depicts the distribution of salt iodine content at different levels in this study.

## Discussion

The prevalence of goiter has decreased gradually over the last 25 years in Iran. According to our previous national study [29], weighted TGR was 5.7% and all subjects had low-grade goiter, and median urinary iodine concentration (UIC) was over 100 µg/dl, indicating that the subjects had adequate iodine intake. The present national survey, which was conducted in 2013–2014, indicated sustainable elimination of IDDs and favorable urinary iodine values in school-aged children of 30 provinces of Iran (Table 1). Since clinical evaluation of low-grade goiter has less validity, in this study urinary iodine rather than goiter prevalence was used as the principal indicator of iodine status.

Median urinary iodine concentration of schoolchildren in the current survey was 161 µg/L. This value was 205 µg/L in 1996, 165 µg/L in 2001 and 140 µg/L in 2007 (Table 2). In 1996, 2 years after mandatory salt iodization in Iran, the median UIC values of schoolchildren were higher than the WHO/UNICEF/ICCIDD optimal levels in many provinces of the country, and in 2001, after 7 years of national salt iodization, despite a change in salt iodine content, the median UIC of Iranian schoolchildren was optimal. The decline in median UIC values from 1996 to 2013 in our study may have been due to changes in dietary habits, particularly in younger people or some other environmental factors. Iodized oil injection during earlier years of salt iodization may also have played a key role in the above optimal levels of median UIC of schoolchildren in 1996. Such a downward trend was also observed between the National Health and Nutrition Examination Surveys 1 and 3 in the USA; however, it finally stabilized at adequate UIC levels in the next survey [30]. This change is particularly important during pregnancy and lactation when low iodine intakes could be accompanied by adverse outcomes in vulnerable populations such as neonates, infants and young children [31].

**Table 2:** Changes in median urinary iodine (MUI) and total goiter rate (TGR) in school-aged children between 1989 and 2013

Year	MUI (µg/L)	TGR^a^ (%)
1989	12–82	68.0
1996	205	54.0
2001	165	9.8
2007	140	5.7
2013	161	Not done

^a^Goiter was assessed by palpation

Since 1996, the mean (± SD) and median salt iodine at the production level have not changed significantly, as current values are 28.2 (± 12.6) and 31.7 ppm, respectively, while corresponding values in 1996 for 278 factory salt samples were 33.8 (± 13.2) and 33.9 ppm, in 2001 for 297 samples 33.2 (± 13.4) and 32.8 ppm, and for 280 samples in 2007, 23.2 (± 13.8) and 34.7 ppm, respectively [32–34]. In 2013, frequency distributions of factory salts with iodine contents of < 20, 20–40 and > 40 ppm were 9, 71 and 20% of samples, whereas in 2007, 2001 and 1996 it was 12, 70 and 18%, 17.2, 54.5 and 28.3% ,and 15.8, 54.7, and 29.5%, respectively. The median iodine contents of household salt sample were 30 ppm in 2013 and 2007 and 32.8 ppm in both 2001 and 1996. The frequency distributions of household table salts showed no significant differences during the last four periods. The iodine contents of < 20, 20–40 and > 40 ppm were 27, 53 and 20% in 2007; 8.3, 71.7 and 20.0% in 2001; and 7.8, 71.9 and 20.3% in 1996, respectively.

Iodine-deficient soil is the major cause of iodine deficiency. Lapses in the monitoring programs and inadequate iodine supplies to the population at risk are the main factors for recurrence of iodine deficiency in a community. Evidence shows IDD relapse in some countries that had previously been successful in controlling IDD; in several countries with well-controlled IDD by USI, control programs have faltered and IDD recurred [35–40]. In some industrialized countries such as Australia and New Zealand, previously thought to be iodine sufficient, IDDs have also relapsed [37, 38]. A study by Zimmermann et al., in an area of endemic goiter in Morocco, showed that changes in thyroid function have occurred after sudden interruption of USI [39]. A survey by Vanderpump indicates that the U.K. population is iodine deficient. In this study, 69% of 810 British girls, aged 14–15 years, had median UIC < 100 µg/L; which is consistent with iodine deficiency according to WHO standards. In addition, 18% had UIC levels below 50 µg/L [40]. These data indicate that IDD control programs are fragile and depend on a strong, long-term commitment from governments, donors, consumers and the salt industry. Monitoring of the indicators is a vital element of an effective and sustained program for the control and elimination of iodine deficiency disorders [41]. An IDD control program is defined by the median urinary iodine concentration (UIC) in school-aged children. If the median UIC is adequate in this group, it is usually assumed iodine intakes of iodized salt are also adequate in the remaining population; therefore based on the results of this study, Iranian populations are consuming adequate iodine, but because iodine requirements sharply increase during pregnancy, universal salt iodization cannot cover this vulnerable group’s iodine needs. Many recent studies have shown that pregnant and lactating women are iodine deficient not only in iodine-deficient regions, but also in iodine-sufficient areas and need at least 150 µg iodine supplementation per day during pregnancy and lactation [42–45]. In another study conducted on 100 pregnant and 84 lactating women, we also found a suboptimal iodine nutritional status in these women [46]. Despite optimal iodine nutrition in Iranian school-aged children, further efforts should be made to understand the importance of, and to guarantee, an adequate iodine intake in pregnant and lactating women.

## Conclusion

A well-organized USI program has ultimately resulted in optimization of UIC and decreased the prevalence of goiter 18 years after USI. The median urinary iodine of schoolchildren was adequate in this survey, as those reported in 1996, 2001 and 2007. According to these criteria, the I.R. of Iran has achieved and maintained a sustainable IDD control program since 1996. The implementation of a sustainable and well-monitored IDD control program needs many effective programmatic steps, in particular its integration into the health network; furthermore, it may also require mandatory iodized salt consumption in certain situations.
